# Study of the antitumour effects and the modulation of immune response by histamine in breast cancer

**DOI:** 10.1038/s41416-019-0636-x

**Published:** 2019-11-21

**Authors:** Melisa B. Nicoud, Helena A. Sterle, Noelia A. Massari, Mónica A. Táquez Delgado, Karina Formoso, María V. Herrero Ducloux, Diego Martinel Lamas, Graciela A. Cremaschi, Vanina A. Medina

**Affiliations:** 1 Laboratory of Tumour Biology and Inflammation, Institute for Biomedical Research (BIOMED), School of Medical Sciences, Pontifical Catholic University of Argentina (UCA), and the National Scientific and Technical Research Council (CONICET), Buenos Aires, Argentina; 2 Laboratory of Radioisotopes, School of Pharmacy and Biochemistry, University of Buenos Aires, Buenos Aires, Argentina; 3 Neuroimmunomodulation and Molecular Oncology Division, Institute for Biomedical Research (BIOMED), School of Medical Sciences, Pontifical Catholic University of Argentina (UCA), and the National Scientific and Technical Research Council (CONICET), Buenos Aires, Argentina; 4 Immunology Department, School of Natural Sciences, National University of Patagonia San Juan Bosco, Chubut, Argentina; 5 Pharmacology and Function of Ionic Channels Laboratory, Institute for Biomedical Research (BIOMED), School of Medical Sciences, Pontifical Catholic University of Argentina (UCA), and the National Scientific and Technical Research Council (CONICET), Buenos Aires, Argentina; 6 Pathology Department, School of Natural Sciences, National University of Patagonia San Juan Bosco, Chubut, Argentina

**Keywords:** Breast cancer, Immunoediting, Targeted therapies, Breast cancer

## Abstract

**Background:**

The aim of this work was to improve the knowledge of the role of histamine in breast cancer by assessing the therapeutic efficacy of histamine and histamine H4 receptor (H4R) ligands in a triple-negative breast cancer (TNBC) model developed in immunocompetent hosts. By using publicly available genomic data, we further investigated whether histidine decarboxylase (HDC) could be a potential biomarker.

**Methods:**

Tumours of 4T1 TNBC cells were orthotopically established in BALB/c mice. Treatments employed (mg kg^−1^): histamine (1 and 5), JNJ28610244 (H4R agonist, 1 and 5) and JNJ7777120 (H4R antagonist, 10).

**Results:**

Increased *HDC* gene expression is associated with better relapse-free and overall survival in breast cancer patients. Histamine treatment (5 mg kg^−1^) of 4T1 tumour-bearing mice reduced tumour growth and increased apoptosis. Although no immunomodulatory effects were observed in wild-type mice, significant correlations between tumour weight and cytotoxic lymphocyte infiltration were detected in H4R knockout mice. H4R agonist or antagonist differentially modulated tumour growth and immunity in 4T1 tumour-bearing mice.

**Conclusions:**

Histamine plays a complex role and stands out as a promising drug for TNBC treatment, which deserves to be tested in clinical settings. HDC expression level is associated with clinicopathological characteristics, suggesting a prognostic value in breast cancer.

## Background

Breast cancer is the most commonly diagnosed neoplasia and the leading cause of cancer-related mortality in women worldwide.^1^ It is a heterogeneous disease, showing different histological types, molecular profiles and clinical responses to therapy.^2^

Triple-negative breast cancer (TNBC) accounts for the 10–20% of breast carcinomas and is characterised by the lack of expression of the oestrogen receptor, progesterone receptor and human epidermal growth factor 2 receptor proteins, and its poor prognosis is associated with a lower rate of relapse-free survival and overall survival. TNBC is the most aggressive subtype of breast cancer with a high proliferative and metastatic potential. As no targeted therapy is available, toxic chemotherapy remains as the only established standard treatment.^3,4^ Therefore, the identification of novel molecular targets for TNBC treatment is urgently needed.

Numerous evidence from both human and murine studies support the key role of the immune system not only in cancer development and progression but also in the response to antitumour treatments.^5–7^ In this regard, a positive correlation between the presence of tumour-infiltrating lymphocytes (TILs) and patients’ survival was demonstrated in different types of tumours, including breast cancer.^8^ In TNBC, in particular, immune escape is principally associated with an immunosuppressive tumour microenvironment.^9^ Therefore, the search for therapeutic strategies that evade immunosuppression, potentiating antitumour immunity and favouring the reduction of metastasis is of utmost importance to improve the survival and quality of life of cancer patients.^8^

Histamine is a biogenic amine with numerous immunomodulatory roles, including modulation of acute and chronic inflammatory and immediate hypersensitivity responses.^10–12^ Histamine effects are mediated by the activation of four different receptor subtypes H1, H2, H3 and H4 receptors (H1R, H2R, H3R and H4R), which belong to the family of seven transmembrane domain G-protein-coupled receptors.^11,12^

Besides the well-documented immune cell responses of histamine, which are mediated by H1R and H2R, new physio- and pathophysiological roles of the latest discovered H4R have been reported over the last decade.^12–16^ The H4R is mainly expressed in cells of the immune system, such as mast cells, basophils, eosinophils, monocytes, dendritic cells, T lymphocytes and natural killer (NK) cells,^12,15^ and its functional expression is demonstrated in different types of tumours.^11,17–20^ H4R was found in human breast cancer tissues and cell lines. The in vivo administration of histamine or H4R agonists diminished the tumour growth of human TNBC developed in immune-deficient nude mice with MDA-MB-231 cells.^21,22^ Importantly, we have recently demonstrated a novel role of H4R in the antitumour immunity of breast cancer,^23^ which could affect the clinical therapeutic outcomes of histamine-related pharmacological compounds.

In addition, many studies have described the expression and activity of the histamine-synthesising enzyme histidine decarboxylase (HDC) in breast cancer patients, but the results are somehow controversial, while its prognostic relevance in breast cancer is still not known.^20^

In the context of the complexity of cancer, effective therapeutics should target the different molecular participants encompassed in a tumour, as well as their specific interactions with the tumour microenvironment. In this regard, histamine, a pleiotropic compound, could be an attractive cancer therapeutic agent. Histamine dihydrochloride administration has been approved in Europe for the treatment of acute myeloid leukaemia, used in combination with the immunotherapeutic agent IL-2, a fact that encourages the study of this biogenic amine as an adjuvant to therapeutic approaches for other cancers.^24,25^

The aim of this work was to improve the knowledge about the role of histamine in breast cancer. By using publicly available genomic data, we first investigated whether HDC could be a potential biomarker, which could correlate with breast cancer prognosis in terms of survival. We further assessed the therapeutic efficacy of histamine and H4R ligands in a preclinical TNBC model developed in immunocompetent host, in which the influence of the immune system in the response to therapeutics could be evaluated.

The results show that histamine plays a complex role and stands out as a promising drug for TNBC treatment, which deserves to be tested in clinical settings.

## Methods

### Chemicals

Histamine (Sigma Chemical Co., Missouri, USA); H4R agonist: VUF8430 (VUF) (Tocris Bioscience, Ellisville, Missouri, USA); JNJ28610244 (JNJ28) (Janssen Research & Development, San Diego, USA). H4R antagonist: JNJ7777120 (JNJ77) (Janssen Research & Development).

### Cell culture

The 4T1 tumour cell line (ATCC CRL-2539) was cultured and maintained in Dulbecco’s Modified Eagle Medium (DMEM) supplemented with 10% (v/v) FBS, 0.3 g L^−^^1^ glutamine, 100 µg ml^−^^1^ streptomycin and 100 U ml^−^^1^ penicillin (all from Gibco BRL, Grand Island, NY, USA).^23^ Cells were maintained at 37 °C in a humidified atmosphere containing 5% CO_2_.

### Cell proliferation assays

For clonogenic assay, cells were left untreated or were treated with 0.01–50 μmol L^−^^1^ of histamine or JNJ28 and/or 10 μmol L^−^^1^ JNJ77 for 5–7 days. We proceeded as previously reported.^26^ The clonogenic proliferation was evaluated by counting the colonies containing 50 or more cells and was expressed as a percentage of the untreated wells.

For the quantification of cellular DNA synthesis, cells were seeded into 12-well plates (2.5 × 10^4^ cells/well), and treated with 10 μmol L^−^^1^ of histamine, JNJ28 or VUF8430 for 48 h. After that, 5-bromo-2′-deoxyuridine (BrdU, 30 mmol L^−^^1^, Sigma) was added into culture medium for 2 h. The incorporation of BrdU to proliferating cells was evaluated as previously described.^26^ The percentage of fluorescent cells was determined by using an Olympus BX50 microscope.

Cell viability was measured with the fluorometric resazurin reduction method (CellTiter-Blue; Promega, Madison, WI, USA). Briefly, 5 × 10^5^ cells/mL were seeded at a final volume of 0.1 mL in 96-well flat-bottom microtiter plates and were treated or not for 48 h with 10 μmol L^−^^1^ of histamine or JNJ28. Fluorescence was determined in a BMG Labtech NOVOstar MicroPlate Reader.

### Apoptosis determinations

Cells were seeded into 12-well plates (2.5 × 10^4^ cells/well), and treated with 10 μmol L^−^^1^ of histamine, JNJ28 or VUF8430 for 48 h. Apoptotic cells were determined by TdT-mediated UTP-biotin Nick End labelling (TUNEL) (Millipore, CA, USA) assay and by staining with Annexin-V FITC (BD Biosciences, San José, CA, USA) by using flow cytometry, both according to the manufacturer’s instructions and previously reported.^19,21^

The cell-permeant, cationic, red-orange fluorescent dye tetramethylrhodamine ethyl ester (TMRE) (Molecular Probes, Life Technologies Corporation, Carlsbad, CA, USA), which is rapidly sequestered by active mitochondria, was used to evaluate the mitochondrial transmembrane potential. Since dead cells become completely depolarised, we analysed live-gated cells to detect the decrease in mitochondrial transmembrane potential, which is associated with apoptosis. Cells were incubated at 37 °C for 30 min in the presence of 40 nmol L^−^^1^ TMRE. They were then harvested after washing with PBS, and analysed by flow cytometry (BD Accuri C6, BDB). The mean fluorescence of untreated cells was set at 100%. CCCP (carbonyl cyanide m-chlorophenyl hydrazone), a mitochondrial oxidative phosphorylation uncoupler, was used as a positive control at a concentration of 20 μmol L^−^^1^ during 30 min.

### Breast cancer model in BALB/c mice

Female H4R knockout (H4R^−/−^) mice were generated as previously described,^27^ and were provided by Janssen Research & Development, LLC (La Jolla, CA, USA) and back crossed to BALB/c background. These animals and the corresponding female wild-type (WT) mice (BALB/c) were bred and kept in ventilated cages at our animal health care facility at 22–24 °C and 50–60% humidity on a 12-h light/dark cycle with food and water available ad libitum.^23^ Animals with an age of 6–8 weeks and an average weight of 20–25 g were used.^23^ All animal protocols were in accordance with recommendations from the National Institute of Health Guide for the Care and Use of Laboratory Animals (NIH Publications No. 8023) and the Guidelines for the welfare and use of animals in cancer research.^23^ All procedures were reviewed and approved by the Institutional Committee for the Care and Use of Laboratory Animals, BIOMED, and are in accordance with the ARRIVE guidelines for reporting experiments involving animals.

To generate solid tumours, 6–8-week-old mice were inoculated orthotopically in the abdominal mammary gland with 1 × 10^5^ syngeneic 4T1 cells in serum-free PBS, as described.^23,28^ Tumour length and width were measured every 2 days by using callipers, and tumour volume was calculated as V = π/6 × length × width^2^.^23^ When tumours became palpable, mice were randomly assigned to the control group (saline treated) or were daily treated (morning) with subcutaneous (s.c.) injections of histamine (1 mg kg^−^^1^ b.w. or 5 mg kg^−^^1^ b.w. diluted in saline), JNJ28 (1 mg kg^−^^1^ b.w. or 5 mg kg^−^^1^ b.w., resuspended in 0.1 N HCl, neutralised with 4 N NaOH and diluted with saline) or JNJ77 (10 mg kg^−^^1^ b.w., diluted in saline) for 15 days. The administration was performed in the animal health care facility on the dorsal flank at concentrations according to previous studies in TNBC and melanoma models developed in immunodeficient mice.^19–21^ The method consists of tenting the skin between the shoulder blades and inserting the needle bevel up in the pocket created. Mice were then killed by cervical dislocation, and tissues were removed and weighted.

To evaluate the combined effect of irradiation and histamine, the tumours from control or histamine- (1 mg kg^−^^1^ b.w.) treated mice were irradiated with a daily dose of 2 Gy for 3 consecutive days as previously described in detail.^29^ Briefly, 1 day after treatment began, animals were irradiated with a 2-Gy dose per day for 3 consecutive days. Mice were anaesthetised with an intraperitoneal injection of a combination of xylazine (10 mg kg^−^^1^) and ketamine (100 mg kg^−^^1^) and fixed on an acryl plate. Tumours were locally irradiated with a ^60^Co γ-radiation source (Teradi 800, at Hospital Municipal de Oncologia “Marie Curie”, Buenos Aires), while other body parts were protected with lead blocks.

### Histochemistry and immunostaining

Tumours and tissues were excised, fixed in 4% (v/v) formaldehyde in PBS (formalin), paraffin-embedded and sliced into 4-μm thick sections to evaluate the histological characteristics on haematoxylin–eosin (H&E)-stained specimens (Biopur diagnostic, Buenos Aires, Argentina). The mitotic index, intratumoural vascularity and the number of lung metastasis were evaluated.

Immunohistochemistry was performed with primary rabbit antihistamine (1:100), rabbit anti-HDC (1:100) and mouse anti-proliferating cell nuclear antigen (PCNA, 1:100) antibodies (see Supplementary Table 1 for more information). The fragmented DNA was detected by using Apoptag^TM^ plus peroxidase in situ apoptosis Detection Kit (Millipore) according to the manufacturer’s instructions.

All these procedures were previously described in detail.^19,21^

Visualisation of samples was performed with an optical microscope Axiolab Carl Zeiss (Germany), and photographs were taken at ×630 magnification with Canon Power Shot G5 camera (Japan).

### Preparation of single-cell suspensions from lymph nodes, spleens and tumours

After removal, lymphoid organs were disrupted through a 1-mm metal mesh. Tumours were minced and digested with 2 mg mL^−^^1^ collagenase type I (Gibco) in serum-free DMEM for 30 min at 37 °C. After centrifugation, the red blood cells were lysed, and the resulting cell suspensions were then filtered through a 40-µm cell strainer (BDB) and resuspended in PBS.^23,28^

### Flow cytometry for immunophenotyping

Single-cell suspensions obtained from tumours, tumour-draining lymph nodes (TDLN), non-draining lymph nodes (LN) and spleens were stained with antibodies against various cell surface markers by using standard staining methods, as previously described.^23,28^ The fluorochrome-conjugated antibodies that were used in the study are shown in Supplementary Table 1. Samples were run on a BD Accuri C6 flow cytometer (BDB), and data were analysed by using the BD Accuri C6 software (BDB). The percentage of tumour-infiltrating lymphocytes (TILs) was determined by forward- and side-scatter properties of these cells. The percentages of tumour-infiltrating CD3^+^, CD4^+^, CD8^+^ and CD3^−^CD49^+^ cells are indicated within the gated population of TILs.

### FoxP3 staining

Single-cell suspensions, prepared as described above, were used for intracellular staining. After CD4 and CD25 surface staining, cells were fixed with Mouse Fixation Buffer (BDB, 51-9006124) and permeabilised with Mouse FoxP3 Permeabilization Buffer (BDB, 51-9006125), following the manufacturer's instructions. Cells were then incubated with the FITC-anti-mouse-FoxP3 antibody (BDB-560408) for 40 min. After washing with PBS, cells were analysed by flow cytometry as described above.^23^

### Cytokine determination

Conditioned medium was obtained after cutting tumours into small pieces and incubating equal quantities of tissue in complete RPMI medium for 48 h. Alternatively, 4T1 cells were treated with histamine or JNJ28, and conditioned medium was obtained after 48 h. Mice interferon (IFN)-γ (BDB-558473), tumour necrosis factor (TNF) (BDB-558480) and interleukin (IL)-10 (BDB- 558300) CBA Flex Sets were used to analyse specific cytokines in a BD Accuri C6 flow cytometer, following the manufacturer's instructions. The results were analysed with FCAP Array Software v3.0 (BDB).^23^

### TCGA and GEO gene expression and survival analysis

UALCAN is an interactive web portal for facilitating tumour subgroup gene expression and survival analyses (http://ualcan.path.uab.edu/).^30^ We used UALCAN analysis to evaluate *HRH4* and *HDC* gene expression levels based on tumour sample type and survival in The Cancer Genome Atlas (TCGA) breast cancer datasets. RNA-seq data obtained from TCGA samples were analysed by using Firebrowse web resource (http://firebrowse.org).^31^

In addition, the web portal Kaplan–Meier plotter (http://kmplot.com/analysis/)^32^ was used to investigate the association between the level of expression of these genes and relapse-free survival (RFS) and overall survival (OS). This online tool allowed the evaluation of the Gene Expression Omnibus (GEO) breast cancer datasets.

Genomic alteration frequency of HDC in breast cancer patients was performed by using TCGA data and CBioPortal web resource (http://www.cbioportal.org/).^33^

GSE62598 microarray dataset from the GEO database was used to evaluate HDC expression on different sources of 4T1 breast cancer model (www.ncbi.nlm.nih.gov/geoprofiles).

### Statistical analyses

The results are presented as the mean ± standard error of the mean (SEM) or the median and interquartile range, as indicated. Student’s *t* test or nonparametric Mann–Whitney *U* test were used for comparisons between two groups. One-way ANOVA followed by Newman–Keuls multiple comparison test or nonparametric Kruskal–Wallis test was used for comparisons between more than two groups. Spearman’s r correlation coefficient and two-tailed significances were determined when appropriate. All statistical analyses were performed with GraphPad Prism version 7.00 (CA, USA). The “*n*” values represent the number of observations or the number of animals used.

## Results

### Association of *HDC* gene expression with survival outcomes in breast cancer patients

**Fig. 1:**
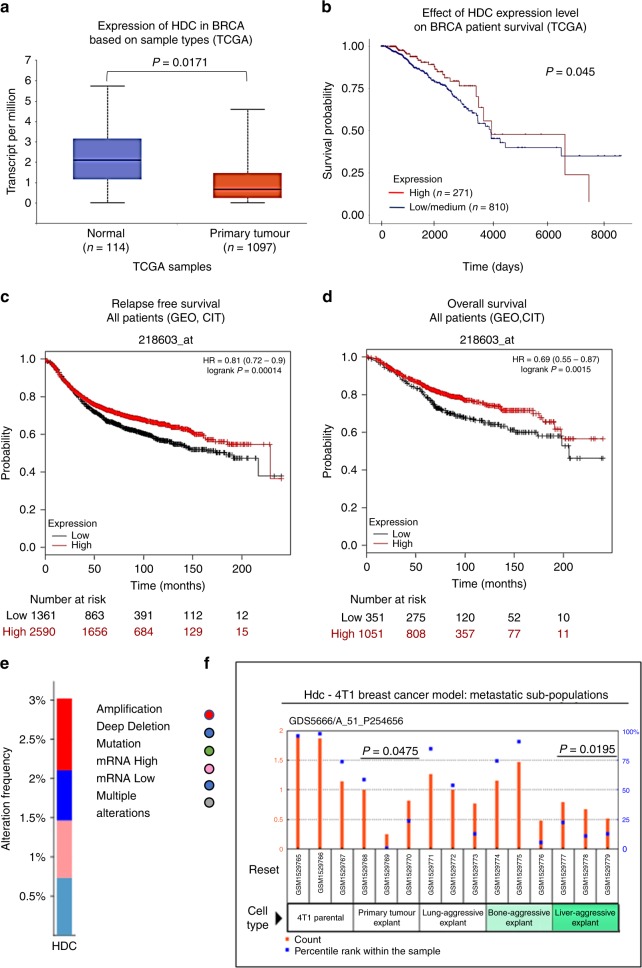
Analysis of histidine decarboxylase (HDC) expression and its association with clinical characteristics of breast cancer patients. **a** Boxplot generated in the UALCAN interactive web resource (http://ualcan.path.uab.edu/), shows relative expression of HDC in transcript per million units (TPM) in paired normal and primary tumour of breast-invasive carcinoma (BRCA) patients. The samples used for the analysis derived from the genomic data of The Cancer Genome Atlas (TCGA) (*T* test). **b** Kaplan–Meier plot generated in the UALCAN with TCGA data demonstrates the association of HDC expression levels with patient overall survival. Samples were categorised into two groups: high expression (red line, with TPM values above the upper quartile), and low/medium expression (blue line, with TPM values below the upper quartile), and the difference was compared by log-rank test (*P* < 0.05 was indicated the cutoff value). **c**, **d** Kaplan–Meier plots generated in the portal web Kaplan–Meier Plotter (http://kmplot.com/analysis/) show the association of HDC expression levels with (**c**) patient relapse-free survival and (**d**) patient overall survival. The samples used for the analysis derived from the Gene Expression Omnibus (GEO) and the *“Cartes d’Identité des Tumeurs* (CIT)” breast cancer (BRCA) datasets. Red line: patients with expression levels above the median; black line: patients with expression levels below the median. The two patient cohorts are compared by a Kaplan–Meier survival plot, and the hazard ratio with 95% confidence intervals and log-rank *P*-value are calculated. Additional clinical parameters are displayed in Supplementary Table 2. **e** Genomic alteration frequencies of HDC in breast cancer patients. The analysis was performed by using CBioPortal database (www.cbioportal.org). **f** GSE62598 microarray dataset from the GEO database was used to evaluate HDC expression on different sources of 4T1 breast cancer model (Mann–Whitney test, *P*-value compared with 4T1 parental cells)

To shed light on the role of histamine in breast cancer progression, we first analysed changes in HDC expression levels between breast cancer and adjacent non-tumour tissues (normal), by using publicly available transcriptomic data. TCGA database and UALCAN web portal revealed that primary tumour samples exhibited reduced levels of HDC when compared with normal tissue (Fig. 1a). Notably, the level of HDC mRNA expression in TNBC tissue was lower than in other breast cancer types (Supplementary Table 2). In addition, the Firebrowse web resource demonstrated a 0.4-fold change of *HDC* expression in tumour vs. normal tissue.

To determine the association between *HDC* gene expression and survival rates in breast cancer patients, large-scale human cancer gene expression independent databases were searched. Kaplan–Meier curves for overall survival of patients with breast cancer were obtained according to the low and high expression levels of *HDC* gene. Patients with high mRNA expression for *HDC* had significantly better overall survival than those in the low/medium expression group (Fig. 1b).

In addition, survival analysis from KM plotter, another publicly available breast cancer microarray database,^32^ showed a significant correlation between high expression levels of *HDC* and a better overall survival and relapse-free survival in all breast cancer patients (Fig. 1c, d). This was also observed in basal-like breast cancer patients, who have the worst prognosis among all subtypes and overlap with TNBC patients in this database (Supplementary Table 2).^34^

The analysis of the available data from cBioPortal revealed a frequency of about 3% in *HDC* alterations in breast cancer, including amplifications and deep deletions (Fig. 1e).

Collectively, these findings suggest that the reduced expression of *HDC* in breast cancer patients might be involved in cancer progression and could be associated with the prognosis of the disease.

### Effect of histamine treatment on 4T1 TNBC tumour growth parameters and antitumour immunity

**Fig. 2:**
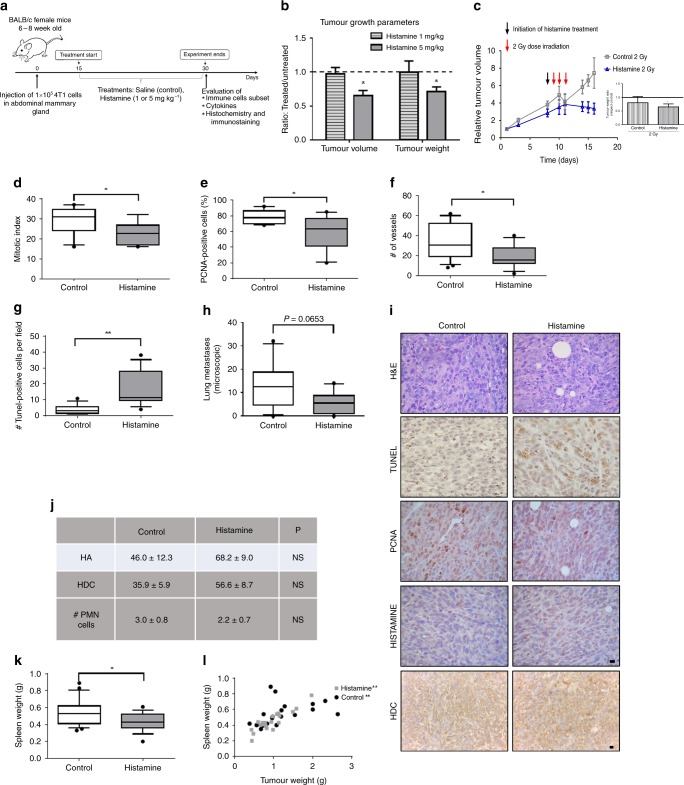
Tumour growth parameters of 4T1 tumour-bearing mice treated with histamine. **a** Experimental design. BALB/c mice were orthotopically inoculated with 4T1 cells and 2 weeks later were treated or not with 1 or 5 mg kg^−^^1^ of histamine. **b** The means ± SEM for the ratio between treated and untreated mice for tumour volume and weight at the end of the experimental period are shown (*T* test, *P < 0.05 with respect to untreated mice). **c** Effect of ionising radiation treatment (three doses of 2 Gy) on untreated and 1 mg kg^−^^1^ histamine-treated mice bearing 4T1 tumours. Tumour growth curves show the relative tumour volume for irradiated control and histamine-treated mice. Inset: Tumour weight at the end of the experimental period, relative to control non-irradiated group (*T* test, *P < 0.05). **d–g** Tumour sections from control (*n* = 10) and 5 mg kg^−^^1^ histamine-treated mice (*n* = 10) were stained with H&E or immunostained with specific antibodies. The box plots represent the median and interquartile range for each experimental group (Mann–Whitney test, *P < 0.05, **P < 0.01). **d** Mitotic index: number of cells with visible chromosomes at ×400 magnification in 5 random fields. **e** Percentage of PCNA-positive cells per field at ×400 magnification in 10 random fields. **f** # of vessels: Number of intratumoural vessels at ×200 magnification in 10 random fields (hotspots). **g** Number of TUNEL-positive cells per field at ×400 magnification in 10 random fields. **h** Number of microscopic metastatic foci covering lungs at ×400 magnification in 10 random fields. Box plots represent the median and interquartile range for each experimental group (Mann–Whitney test). **i** Representative images for H&E staining, TUNEL-positive cells and PCNA, histamine and HDC-positive immunostaining of paraffin-embedded 4T1 tumours (×630 and ×400 original magnification, scale bar = 20 µm). **j** Tumour sections from control and 5 mg kg^−^^1^ histamine-treated mice were stained with specific antibodies against histamine (HA) or histidine decarboxylase (HDC). The results representing the means ± SEM of the percentage of positive cells per field at ×400 magnification in 10 random fields are shown (*t* test). # number of polymorphonuclear neutrophils (PMN), granulocytes with visible segmented nucleus at ×400 magnification in 5 random fields is depicted (*t* test). **k** Spleen weight of control and histamine-treated 4T1 tumour-bearing mice (Mann–Whitney test, *P < 0.05 vs. control). **l** Spearman correlation of spleen weight vs. tumour weight for control mice (*r:* 0.6508, **P = 0.0019; *n* = 20) and mice treated with histamine (*r*: 0.7860, **P < 0.0001; *n* = 20)

We next evaluated the effect of histamine treatment in a murine TNBC experimental model developed with 4T1 cells. From the GSE62598 dataset of GEO database, we assessed the *HDC* expression in 4T1 TNBC. This histamine-synthesising enzyme seemed to be downregulated in tumour explants and liver-aggressive explants compared with 4T1 parental cells (Fig. 1f). We additionally investigated the expression of HDC and intracellular histamine in 4T1 tumours. The presence of intracellular histamine and HDC was observed in all samples (Fig. 2i, j). However, the levels of HDC protein expression were more variable among specimens (Fig. 2i).

We evaluated the effect of histamine administration in vivo, on tumours developed in BALB/c mice by orthotopic injection of 4T1 TNBC cells (Fig. 2a). Two different concentrations of histamine (1 and 5 mg kg^−^^1^) were used, according to previous studies in TNBC and melanoma models developed in immunodeficient mice.^19–21^

However, only histamine at 5 mg kg^−^^1^ significantly reduced the tumour volume and weight (0.65 ± 0.06 vs. 0.91 ± 0.06 g, *P* < 0.05) (Fig. 2b). Although histamine at 1 mg kg^−^^1^ concentration was not able to decrease tumour growth as a single agent, it significantly enhanced the therapeutic effect of radiation, by reducing exponential tumour growth and inducing a significant decrease in the tumour weight (Fig. 2c).

For further experiments histamine was used at a concentration of 5 mg kg^−^^1^. Histopathological analysis of all tumours demonstrated the presence of undifferentiated adenocarcinoma cells with a high grade of nuclear polymorphism (Fig. 2i). In addition, it showed that tumours from histamine-treated mice presented a reduced mitotic index (Fig. 2d) and a lower percentage of PCNA-positive cells (Fig. 2e, i). In addition, histamine significantly increased the number of apoptotic cells compared with the untreated animals (Fig. 2g, i). Treatment of 4T1-bearing mice with histamine reduced the number of intratumoural vessels (Fig. 2f) and non-significantly decreased the number of microscopic lung metastases (Fig. 2h, *P* = 0.0683). In line with the histamine-induced inhibition of tumour growth, histamine-treated animals also showed reduced splenomegaly (Fig. 2k). In both experimental groups, the splenic weight was correlated with tumour weight (Fig. 2l), as it has been previously described for this TNBC experimental model.^35^

**Fig. 3:**
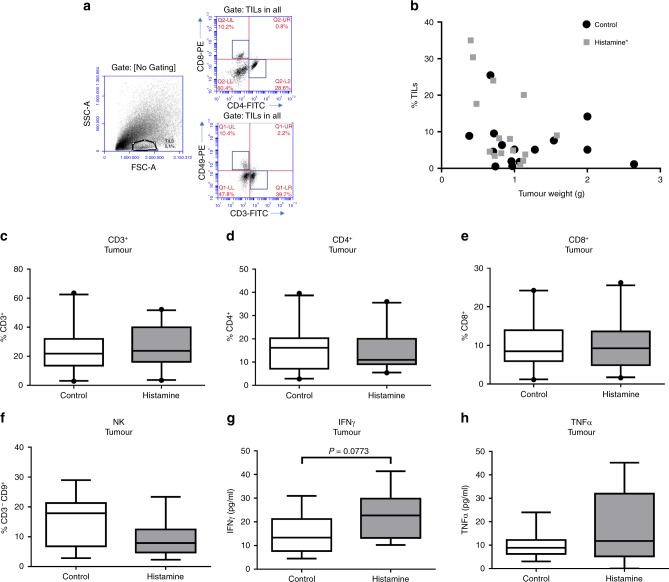
Distribution of tumour-infiltrating immune cell subsets in control and histamine-treated mice. Mice bearing 4T1 tumours were treated with saline (control) or histamine (5 mg kg^−^^1^). **a** Gating scheme for the flow cytometric analysis of tumour-infiltrating lymphocytes (TILs). Representative data of single-cell suspensions from enzymatically dissociated tumours, labelled with antibodies according to procedures described in the “Methods” section. Numbers represent the percentage of cells in the respective gate. **b** The percentage of TILs (% TILs), determined by flow cytometric analysis of forward vs. side scatter, was correlated with tumour weight for each experimental group. Spearman correlation for control mice (*P* > 0.05; *n* = 15) and mice treated with histamine (correlation coefficient, *r*: –0.4821, *P < 0.05; *n* = 15). **c–f** Tumour cell suspensions were labelled with specific antibodies, and the percentage of each subset was determined within the TIL-gated population: **c** CD3-FITC: T lymphocyte marker (*n* = 20 control; *n* = 21 histamine), **d** CD4-FITC: T helper lymphocyte marker (*n* = 20 control; *n* = 21 histamine), **e** CD8-PE: T cytotoxic lymphocyte marker (*n* = 20 control; *n* = 21 histamine), **f** CD49-PE and CD3-FITC: NK markers (*n* = 14 control; *n* = 14 histamine). **g**, **h** Cytokine concentrations in tumour-conditioned medium (*n* = 12 control; *n* = 10 histamine). The box plots represent the median and interquartile range for each experimental group (Mann–Whitney test)

In view of the critical role of immunity in the tumour microenvironment and the pivotal immunomodulatory role of histamine, the inflammatory tumour infiltrate was next investigated. To this end, TILs were evaluated by FACS according to the gating strategy depicted in Fig. 3a. A non-significant higher percentage of TILs was observed in tumours from the histamine-treated group [median 8.04 (3.79–20.08), *n* = 15] compared with the control group [median 5.13 (1.79–8.87), *n* = 15, *P* > 0.05, Mann–Whitney test], and a negative correlation was observed between TILs and tumour weight only on histamine-treated animals (Fig. 3b). The analysis of the distribution of the tumour-infiltrating immune cell subsets within the TIL-gated population was next performed and showed no significant changes in the percentage of CD3^+^, CD4^+^ or CD8^+^ T lymphocytes, or NK cells between treated and untreated animals (Fig. 3c–f). Treg cell population was not detected in tumours. Since myeloid cells are major components of tumour microenvironment and are involved in cancer progression,^8^ they were further investigated. The results showed no significant differences neither in the percentage of tumour-infiltrating CD11b^+^Gr1^+^ myeloid-derived suppressor cells (MDSC) nor in F4/80^+^ macrophages when histamine-treated and untreated mice were compared (Supplementary Fig. 1). Likewise, histopathological analysis revealed no changes in intratumoural neutrophils, evaluated by their morphological characteristics (Fig. 2j).

The analysis of cytokine production in the conditioned medium of tumours showed no differences in the levels of TNFα (Fig. 3g). However, a non-significant increase in IFNγ levels was detected in the conditioned medium from tumours derived from histamine-treated mice (Fig. 3h).

The effect of the treatment with histamine (5 mg kg^−^^1^) on the distribution of immune cells in the spleens, non-draining lymph nodes (LN) and tumour-draining lymph nodes (TDLN) of 4T1 tumour-bearing mice was additionally evaluated. No significant differences were observed regarding the percentages of CD4^+^ or CD8^+^ T lymphocytes nor NK cells in the spleen or in the lymph nodes of both animal groups (Supplementary Figs. 2, 3, 4). The cytotoxic activity of splenic NK cells and CD8^+^ T cells from spleens, TDLN and LN was further investigated, but no changes were detected between the control and histamine-treated group (Supplementary Figs. 3 and 4). A non-significant reduction of the percentage of CD4^+^CD25^+^FoxP3^+^ Treg cells was observed in the spleens and LN of animals treated with histamine (5 mg kg^−^^1^) compared with the control group (Supplementary Fig. 2).

In line with these results, no significant correlations between tumour weight and cytotoxic lymphocyte infiltration, cytotoxic T cells (CD8^+^) and NK cells, were observed in untreated or histamine-treated tumour-bearing wild-type mice. Surprisingly, negative correlations between the percentage of tumour-infiltrating NK cells, CD8^+^ T lymphocytes or activated CD8^+^ CD44^+^ T lymphocytes and tumour weight were detected only in histamine-treated H4R knockout (KO) mice. In addition, a significant positive correlation between the ratio of CD4^+^/CD8^+^ T cells and tumour weight was observed upon histamine treatment in H4R-KO mice, further demonstrating the crucial role of H4R in immunosurveillance (Table 1).

**Table 1:** Correlation coefficient (*r*) value of tumour weight (g) vs. immune cells subset percentage in tumours of wild-type and H4R-KO mice

Correlations	Wild-type mice		H4R-KO mice	
	Control	Histamine	Control	Histamine
% NK cells	*N*: 22	*N*: 16	*N*: 14	*N*: 10
	*P*: ns	*P*: ns	*P*: ns	*P* = 0.0544
				*r* = –0.6364
% CD8^+^ T cells	*N*: 33	*N*: 21	N: 17	*N*: 10
	*P*: ns	*P*: ns	*P*: ns	*P* = 0.0126
				*r* = –0.7697
% CD8^+^ CD44^+^ T cells	*N*: 10	*N*: 11	*N*: 10	*N*: 9
	*P*: ns	*P*: ns	*P*: ns	*P* = 0.0589
				*r* = –0.6667
CD4^+^/CD8^+^ T cells	*N*: 31	*N*: 21	*N*: 17	*N*: 10
	*P* = 0.0069	*P*: ns	*P*: ns	*P* = 0.0037
	*r* = 0.4752			*r* = 0.8424

Tumour cell suspensions from control and histamine- (5 mg kg^−^^1^) treated mice were labelled with specific antibodies: CD49-PE and CD3-FITC: NK markers, CD8-PE: cytotoxic T lymphocyte marker and CD44-FITC: lymphocyte activation marker. The percentage of each subset and the CD4^+^/CD8^+^ T lymphocyte ratio was correlated with tumour weight. Spearman correlation coefficient (*r*) was calculated for each experimental group

### Effect of H4R ligands on 4T1 TNBC tumour growth parameters and antitumour immunity

**Fig. 4:**
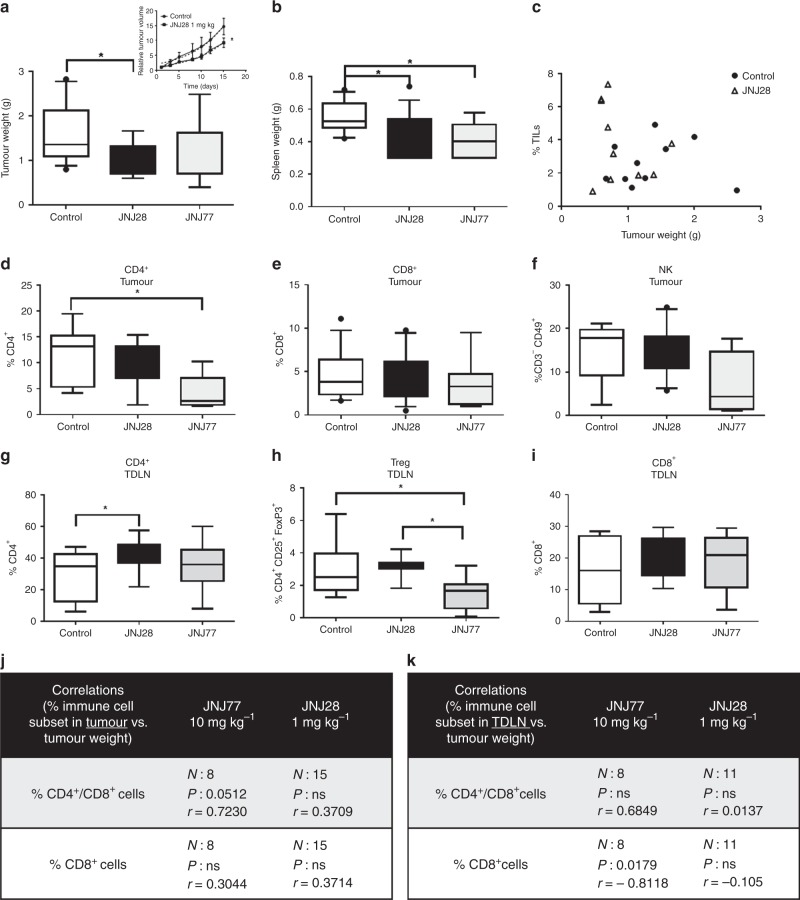
Tumour growth parameters of 4T1 tumour-bearing mice treated with H4R ligands. 4T1 tumour-bearing mice were left untreated (control) or were treated with 1 mg kg^−^^1^ of the H4R agonist JNJ28610244 (JNJ28) or 10 mg kg^−^^1^ of the H4R antagonist JNJ7777120 (JNJ77) for 15 days. **a** Tumour weight at the end of the experimental period (*n* = 12 control; *n* = 14 JNJ28; *n* = 9 JNJ77). Inset: Tumour growth curves show the relative tumour volume for control and JNJ28-treated mice. **b** Spleen weight of control (*n* = 12), JNJ28 (*n* = 14) and JNJ77-treated (*n* = 9) 4T1 tumour-bearing mice. **c** The percentage of tumour-infiltrating lymphocytes (% TILs), determined by flow cytometric analysis of forward vs. side scatter, was correlated with tumour weight for control (*n* = 10) and JNJ28-treated (*n* = 10) mice. Spearman correlation (*P* > 0.05). **d–k** Tumour and tumour-draining lymph node (TDLN) cell suspensions were labelled with specific antibodies: **d**, **g** CD4-FITC: T helper lymphocyte marker (*n* = 7 control; *n* = 9 JNJ28; *n* = 7 JNJ77 for intratumoural. *n* = 13 control; *n* = 15 JNJ28; *n* = 10 JNJ77 for TDLN), **e**, **i** CD8-PE: T cytotoxic lymphocyte marker (*n* = 13 control; *n* = 15 JNJ28; *n* = 8 JNJ77 for intratumoural. *n* = 10 control; *n* = 11 JNJ28; *n* = 8 JNJ77 for TDLN), **f** CD49-PE and CD3-FITC: NK markers (*n* = 8 control; *n* = 10 JNJ28; *n* = 4 JNJ77 for intratumoural). **h** CD4-FITC, CD25-APC and FoxP3-FITC: regulatory T cells (*n* = 9 control; *n* = 11 JNJ28; *n* = 8 JNJ77 for TDLN). Box plots represent the median and interquartile range for each experimental group (Kruskal–Wallis test, *P < 0.05). Spearman correlation of **j** the percentage of tumour-infiltrating immune cell subsets vs. tumour weight and **k** the percentage of immune cell subsets in TDLN vs. tumour weight for mice treated with JNJ28 and JNJ77

To improve our knowledge on the role of H4R in histamine-mediated responses, the effect of an H4R agonist (JNJ28) and an H4R antagonist (JNJ77) was evaluated in vivo by employing the 4T1 breast cancer model. Only 1 mg kg^−^^1^ of JNJ28, but not 5 mg kg^−^^1^ (data not shown), exhibited an antitumour effect, significantly reducing the tumour volume and its weight at the end of the experiment, together with a decrease in spleen weight (Fig. 4a, b). However, at this concentration, no significant differences were observed in the number of TILs or immune cell subset distribution in tumours of JNJ28-treated mice compared with untreated ones (Fig. 4c–f). The evaluation of the immune cell subsets in secondary lymphoid organs, however, indicated that JNJ28 administration increased the percentage of CD4^+^ T cells in TDLN compared with untreated animals (Fig. 4g).

On the other hand, dissimilar effects were observed upon administration of the H4R antagonist JNJ77, at a concentration routinely employed. JNJ77 treatment non-significantly reduced tumour weight and spleen weight, while it significantly decreased the percentage of tumour-infiltrating CD4^+^ T lymphocytes and further reduced the percentage of Tregs in TDLN (Fig. 4a, b, d, h).

None of the compounds significantly altered the percentage CD8^+^ T cells or NK cells within the tumour infiltrate or TDLN (Fig. 4e, f, i).

Interestingly, only in JNJ77-treated mice, the percentage of CD8^+^ T cells in TDLN negatively correlated with tumour weight, and the intratumoural ratio CD4^+^/CD8^+^ T cells positively correlated with tumour weight (Fig. 4j, k). No important adverse events were observed upon treatment with any compound.

### Direct antiproliferative and proapoptotic effects of H4R agonists on 4T1 cells

**Fig. 5:**
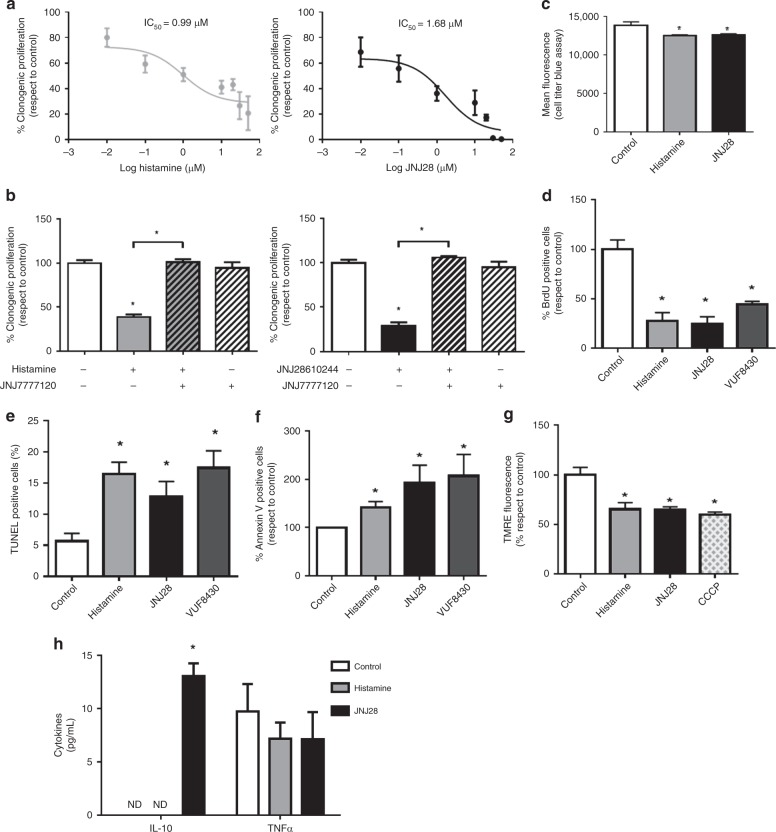
Effect of histamine and H4R agonists on in vitro 4T1 cell growth. **a** Cells were left untreated (control) or were treated with histamine (0.01–50 μmol L^−^^1^) or JNJ28 (JNJ28) (0.01–50 μmol L^−^^1^) for 7 days. Proliferation was evaluated by the clonogenic assay. The half-maximal inhibitory concentration (IC_50_) is indicated for histamine and JNJ28. **b** Cells were treated with histamine (10 μmol L^−^^1^), JNJ28 (10 μmol L^−^^1^) and/or JNJ77 (10 μmol L^−^^1^) for 7 days. Proliferation was evaluated by the clonogenic assay. **c**–**h** Cells were left untreated (control) or were treated with 10 μmol L^−^^1^ of histamine, JNJ28 or VUF8430 for 48 h. **c** The cell viability was evaluated by Cell Titer Blue Assay. **d** BrdU incorporation assay. **e** Percentage of TUNEL-positive cells. **f** Percentage of Annexin-V positive cells. **g** Measurement of mitochondrial transmembrane potential by TMRE staining. CCCP (carbonyl cyanide m-chlorophenyl hydrazone), a mitochondrial oxidative phosphorylation uncoupler, was used as a positive control. **h** Cytokine concentrations in 4T1 cells-conditioned medium. Error bars represent the mean ± SEM (*n* = 3–5 independent experiments). (*P < 0.05, ANOVA and Newman–Keuls multiple comparison test)

With the aim of trying to discern the contribution of a direct action of the compounds on tumour cells, we performed in vitro studies to evaluate the effect of H4R agonists on the proliferative and apoptotic responses of 4T1 cells, which are known to express H4R at the mRNA and protein levels.^23^ The results demonstrated that histamine regulated the clonogenic proliferation of 4T1 cells in a dose-dependent manner, exhibiting an IC_50_ of 0.99 µmol L^−^^1^ (Fig. 5a). H4R agonist JNJ28 showed an IC_50_ of 1.68 µmol L^−^^1^ (Fig. 5a). The antiproliferative effects of histamine and JNJ28 were blocked by the addition of the specific H4R antagonist JNJ77 at the concentration of 10 µmol L^−^^1^ (Fig. 5b).

As expected, cell viability was significantly decreased in cells that were treated with histamine (10 µmol L^−^^1^) and JNJ28 (10 µmol L^−^^1^), as it was evidenced by lower mean fluorescence intensities than untreated cells in the Cell Titer Blue Assay (Fig. 5c). The antiproliferative role of H4R was also confirmed with the BrdU assay in cells treated with histamine, JNJ28 or VUF8430, another H4R agonist (Fig. 5d). In addition, the treatment of 4T1 cells with histamine and both JNJ28 and VUF8430 increased the percentage of apoptotic cells, evaluated by TUNEL assay and flow cytometric analysis of Annexin-V staining (Fig. 5e, f). Accordingly, treatment with histamine and JNJ28 reduced mitochondrial transmembrane potential evaluated by TMRE staining (Fig. 5g). In agreement with these results, high *HRH4* gene expression in breast tumours is associated with a better patient relapse-free survival (Supplementary Table 2).

Finally, we evaluated if H4R agonists can regulate the pattern of cytokine secretion by 4T1 cells. The conditioned media obtained after 48 h of culture showed undetectable levels of IFNγ in untreated as well as in histamine- or JNJ28-treated cells. Likewise, TNFα secretion levels were similar in all experimental conditions. However, a significant increase in IL-10 levels was detected only in JNJ28-treated 4T1 cells (Fig. 5h).

## Discussion

The triple-negative tumours are undoubtedly one of the most relevant subgroups of breast cancer given their lack of targeted therapies, their aggressive clinical behaviour and poor prognosis.^4,36,37^ Identifying novel potential biomarkers for early diagnosis, prognostic determination or targeted therapy are of utmost importance to improve TNBC patient outcomes. In this sense, TCGA and other publicly available databases, such as GEO, produce a rich and invaluable genomic data generation, which notably contributes to accelerate the understanding of the molecular basis of cancer and to improve drug development and treatment strategies.

In the present work, we first analysed the expression of the histamine-synthesising enzyme HDC in human breast cancer tissues. Genomic data from TCGA show that *HDC* is downregulated in breast cancer in comparison with normal tissue. Samples from the most aggressive cancer subtypes, such as TNBC, exhibited the lowest *HDC* gene expression levels. Further supporting the relevance of histamine in cancer progression, the results demonstrated that higher HDC expression predicted longer overall survival. In addition, higher expression of *HDC* was significantly associated with improved disease-free survival in all breast cancer patients as well as in TNBC patients, suggesting that histamine may reduce or delay metastatic breast cancer. In this line, elevated *HDC* gene expression was associated with improved survival outcomes in colorectal cancer patients.^38^ We could also describe genomic alterations of *HDC* in breast cancer samples. In this regard, a study in the Chinese Han population showed that polymorphisms of *HDC* gene were associated with breast cancer, further highlighting the clinical relevance of HDC in this disease.^39^

Thus, our results suggest that high HDC expression might predict a better clinical outcome, reducing the risk of cancer relapse and might be a novel prognostic marker for breast cancer progression. Further immunohistochemical studies in a large number of tumour samples are needed to validate the use of HDC as a biomarker, which could complement routine histopathological analysis. Considering that the HDC activity is regulated at different levels, the presence of HDC proteolytic variants and other post-translational modifications should be considered in the analysis.^12^

Preclinical research from different groups has shown the therapeutic efficacy of histamine and specific H4R agonists in different experimental cancer models. But these studies have been mainly performed in immunodeficient hosts, in which the effect of the immune system in response to therapeutics could not be contemplated. Considering the key role of histamine and H4R in immunomodulation, it is necessary to corroborate the therapeutic benefit globally, by considering the role of immune response in the outcome.^40^

Given the relationship between the histamine-forming enzyme and the patient’s prognosis, the effect of histamine systemic treatment on tumour growth and in the immune tumour microenvironment as a whole was explored in a TNBC syngeneic model developed in immunocompetent mice. The 4T1 tumour model resembles human TNBC as it leads to metastasis formation. It is commonly used for the preclinical evaluation of anticancer therapies on tumour development and the immune system.^28,41^

Histamine treatment leading to a dose-dependent effect, was the highest concentration that has been employed that resulted to be the therapeutically effective one. Histamine administration significantly reduced 4T1 tumour size and weight. This effect was associated with a reduction in the proliferative potential of tumours and an increase in the apoptotic cell death, together with a higher infiltration of lymphocytes. In line with these results, we have previously demonstrated similar effects in a human TNBC model developed in nude mice in which histamine administration at 5 mg kg^−^^1^ significantly increased the median survival and tumour apoptosis, while it reduced tumour cell proliferation.^21^ In both murine and human TNBC models, histamine reduced the neovascularisation of the tumour microenvironment. The administration of histamine also diminished angiogenesis in a human model of melanoma.^19^ All 4T1 tumours expressed similar levels of HDC and exhibited intracellular histamine.

Histamine being a pleiotropic mediator of inflammatory responses and a regulator of immune cell functions,^12^ we further evaluated the effect of histamine treatment on immune cell subset distribution. The nature of the developed immune responses and TILs determines the outcome of antitumour immunity.^42,43^ The infiltrating cytotoxic cells, mainly cytotoxic CD8^+^ T lymphocytes and NK cells, are ultimately responsible for killing the cancer cells and controlling the tumour growth. Thus, the presence of immunosuppressive cells, such as Tregs and MDSC, is usually associated with worse prognosis.^5,44^ Unexpectedly, the therapeutic concentration of histamine neither modified the distribution of immune cell subsets nor significantly altered the activity of CD8^+^ T lymphocytes and NK cells in 4T1 tumours. In agreement with previous studies, CD11b^+^Gr^+^ MDSC and F4/80^+^ macrophages were the predominant 4T1 tumour-infiltrating immune cells,^45^ but no differences were observed upon histamine treatment in this myeloid linage cell infiltration.

Histamine treatment non-significantly increased IFNγ production in tumours, while no changes were observed in other important cytokines related to cancer progression, such as TNFα.^46,47^ IFNγ is involved in the promotion of hosts’ antitumour immunity and in the cancer elimination (immunosurveillance) process, considering its antiproliferative and proapoptotic effects.^5,6,48^ In vitro, 4T1 cells produced no detectable levels of this cytokine (data not shown). Although CD8^+^ T cells and NK cells are good candidates for being the intratumoural source of this cytokine, other immune cells, including B lymphocytes, macrophages, dendritic cells and granulocytes,^45^ could be responsible for this response.

In agreement with the present results, in a murine lymphoma model developed with EL-4 cells, histamine treatment reduced tumour growth while it induced intratumoural accumulation of maturated dendritic cells.^49^ In addition, enhanced inflammation and higher tumour burden at mucosal sites (intestine and skin) were shown in models of chemically induced carcinogenesis developed in HDC knockout mice compared with wild-type animals. These effects were reverted by histamine administration.^50^ In this line, the histamine-producing probiotic hdc+ *Lactobacillus reuteri* decreased the number and size of colon tumours in HDC KO mice and reduced the gene expression of proinflammatory cancer-associated cytokines, indicating that histamine can suppress colorectal tumorigenesis and the severity of inflammation-associated colon cancer.^38^ On the other hand, endogenous histamine contributed to the tumour growth in a model of breast cancer developed in HDC knockout mice by suppressing the antitumour immunity.^51^

Therefore, different histamine levels and altered histamine metabolism at the tumour site, distinct tumour microenvironments and characteristics and the differential expression of histamine receptors may determine the outcome of the disease.

It is worth noting that histamine was able to potentiate the radiation therapeutic effect even at a lower concentration (1 mg kg^−^^1^), suggesting that it could be an attractive agent to be used also in combination therapies. In this regard, histamine is being administered as an adjuvant to immunotherapy with IL-2 for the treatment of patients with metastatic melanoma and acute myeloid leukaemia, demonstrating clinical benefits.^24,52^

Considering that 4T1 cells express H4R, here we show evidence for a direct cytotoxic effect of histamine in vitro through tumour cell-intrinsic mechanisms involving activation of H4R, which could contribute to the antitumour and proapoptotic effects described above with less contribution of immune-mediated effects.

Nevertheless, we cannot discard a histamine-induced modulation of the immune cell subset distribution during tumour development. However, the effect of histamine on immunocompetent cells could be the result of the histamine concentration reached locally and the affinity constant of the different histamine receptors expressed in the cells that results in the activation of receptors with distinct biologic activities. In accordance with this hypothesis, we have recently demonstrated the involvement of H4R in the tumour immunity in 4T1 breast cancer, showing immunosuppressive effects. Thus, endogenous histamine in H4R- deficient mice produced a reduction of the tumour size and decreased percentages of CD4^+^ T cells and Tregs (CD4^+^CD25^+^FoxP3^+^) in TDLN compared with wild-type mice.^23^ To further reinforce the important role of H4R in immunosurveillance, the present findings show negative correlations between the tumour-infiltrating cytotoxic subsets, CD8^+^ T lymphocytes and NK cells, and the tumour weight, which were only observed in histamine-treated H4R-KO mice.

To keep deciphering the role of H4R in breast cancer, in the present work, we investigated the effect of the systemic administration of the H4R agonist JNJ28 and the H4R antagonist JNJ77 in the same TNBC model developed in wild-type mice.

It is important to point out that the concentration of JNJ28 to a large extent determined the outcome of its therapeutic and immunomodulatory effects in vivo. The lowest concentration (1 mg kg^−^^1^) of JNJ28 slightly, but significantly, reduced tumour size and increased the percentage of CD4^+^ T cells in TDLN.

In contrast, a higher concentration (5 mg kg^−^^1^) of this agonist induced no changes in tumour growth (data not shown) probably because of an immunosuppressive effect on the tumour microenvironment. We have previously shown that JNJ28 (5 mg kg^−^^1^) increased IL-10 and decreased IFNγ levels in 4T1 tumours, this was accompanied by an increase in the percentage of Tregs in TDLN.^23^ In agreement with these results, the treatment with JNJ77 reduced the percentage of tumour-infiltrating CD4^+^ T cells and Tregs in TDLN, similar to the response observed in H4R-KO mice.

Taken together, these results suggest an immunosuppressive effect of H4R in immune cells. This could partially explain the effects observed when 4T1 cells were treated with the highest concentration of H4R agonist, which may in the balance prevail over the direct antiproliferative effect on tumour cells, and thus it may be a determinant for the non-effective therapeutic outcome.

On the other hand, in both in vitro studies as well as in vivo studies performed in immunodeficient hosts, where the role of the immune system in the response to anticancer treatments could not be evaluated, the treatment with H4R agonists showed very promising outcomes.^18–21,40,53,54^ In line with this, the results indicate that elevated *HRH4* gene expression was associated with improved relapse-free survival outcomes in all breast cancer patients.

The higher antitumoural and antimetastatic effects of histamine treatment compared with JNJ28 administration could be associated with the multifaceted action of histamine on different receptors and cell types, which on the one hand balanced antitumour immunity and on the other hand, by acting directly through the H4R on 4T1 tumour cells, reduced proliferation. However, the precise mechanism underlying these effects deserves further investigation.

These results highlight the complexity of cancer disease and the critical interplay between tumour cells and host immune response that determines the clinical therapeutic outcomes.

Based on the presented evidence, combining patients’ survival analysis and in vitro and in vivo studies in TNBC model, we conclude that HDC may be a potential prognostic biomarker in breast cancer that could complement routine histopathological analysis. Histamine is a promising drug to be used as a single or combination therapy for TNBC treatment, which deserves to be tested in prospective clinical trials. The fact that histamine has already been approved to be used in humans reduces the gap between experimental work and the potential clinical application.

## Supplementary information

Supplementary material

## Data Availability

All data generated or analysed during this study are included in this article.
