# Supplementary material for: Study of the antitumour effects and the modulation of immune response by histamine in breast cancer
Source: Br J Cancer. 2019 Nov 21;122(3):348–60. doi: 10.1038/s41416-019-0636-x (PMC7000401; doi:10.1038/s41416-019-0636-x)
Supplement: Supplementary file 1 — Supplementary material [file 41416_2019_636_MOESM1_ESM.pdf]

Supplementary Table 1

Table 1: Antibody reagents used for experiments

Reagent	Supplier	Cat.no/lot no.	Isotype	Clone
**Alexa Fluor® 647 Mouse anti-T-bet**	BD Pharmingen™	561267/8078512	Mouse IgG1, κ	O4-46
**Anti-BrdU (Mouse)**	BD Pharmingen™	555627/6263519	Mouse IgG1, κ	3D4
**Anti-HDC**	Euro-Diagnostica	B 260-1		
**Anti-HNMT**	Novus Biologicals	NBP1-89495	IgG	
**Anti-HRH4 (Rabbit)**	Abcam	Ab97487/GR16863-20	IgG	
**Anti-Mouse CD16/32 (Fc Block)**	BD Pharmingen™	553142/6266549	Rat IgG2b κ	2.4G2
**Anti-PCNA**	Dako Cytomation	M0879/00083603	IgG2a, κ	PC10
**APC Rat anti-Mouse CD25**	BD Pharmingen™	558643/8142691	Rat(LEW) IgG2b, κ	3C7
**APC Rat anti-Mouse CD4**	BD Pharmingen™	553051/6067772	DA/HA IgG2a, κ	RM4-5
**APC Rat anti-Mouse CD44**	BD Pharmingen™	561862/7334629	Rat IgG2b, κ	IM7
**FITC Rat Anti-Mouse CD4**	BD Pharmingen™	557303/6032902	Rat(LEW) IgG2b, κ	GK1.5
**FITC Rat Anti-Mouse CD3**	BD Pharmingen™	561798/5295522	Rat(SD) IgG2b, κ	17A2
**FITC Rat Anti-Mouse Ly-6G and Ly6C**	BD Pharmingen™	553126/6202975	Rat IgG2b, κ	RB6-8C5
**PE Rat Anti-Mouse CD49b**	BD Pharmingen™	561066/7271756	Rat(LEW) IgM, κ	DX5
**PE Rat Anti-Mouse CD8a**	BD Pharmingen™	553032/6110823	Rat(LOU) IgG2a, κ	53-6.7
**PE Rat Anti-Mouse F4/80**	BD Pharmingen™	565410/6063689	Rat(WI) IgG2a, κ	T45-2342
**PE Rat Anti-Mouse CD19**	BD Pharmingen™	557399/6054682	Rat(LEW) IgG2a, κ	1D3
**PE Rat Anti-Mouse CD11b**	BD Pharmingen™	557397/6092882	Rat(DA) IgG2b, κ	M1/70
**PE Rat Anti-Mouse Foxp3**	BD Pharmingen™	560408/6190826	Rat IgG2b	MF23
**PE Mouse Anti-GATA3**	BD Pharmingen™	560074/8100697	Mouse(BALB/c) IgG1, κ	L50-823

T-Bet (T-box gene expressed in T cells): transcription factor associated with T helper 1 cell differentiation; **BrdU**: 5-bromo-2′-deoxyuridine; **HDC**: histidine decarboxylase; **HNMT**: Histamine N-Methyltransferase; **HRH4**: histamine receptor H4; **CD16/32**: blocks non-antigen-specific binding of immunoglobulins to the Fc receptors; **PCNA**: Proliferating cell nuclear antigen; **CD25** (IL-2 receptor a chain): CD4+ thymocytes marker; **CD4**: T helper lymphocytes marker; CD44: T lymphocytes activated marker; **CD3**: T lymphocytes marker, **Ly-6G** and **Ly6C**: myeloid differentiation antigen Gr-1 marker; **CD49b**: natural killer cells marker; **CD8**: T cytotoxic lymphocytes marker; **F4/80**: macrophage marker; **CD19**: B lymphocytes; **CD19b**: myeloid cells marker; **FoxP3** (forkhead box P3): T regulatory cell marker, **GATA3** (GATA binding protein 3): transcription factor associated with T helper 2 cell differentiation.

Supplementary Table 2

Table 2: Association between histidine decarboxylase (HDC) and histamine H4 receptor (HRH4) expression and clinical characteristics of breast cancer patients

	Clinical Parameters	ALL PATIENTS				TNBC PATIENTS			
**HDC**	**Expression (TPM)**	**Normal tissue**	**Primary tumor**	**P**		**Normal tissue**	**TNBC**	**P**	
		**N:** 114<br>**UQ:** 3.142<br>**M:** 2.096<br>**LQ:** 1.160	**N:** 1097<br>**UQ:** 1.446<br>**M:** 0.67<br>**LQ:** 0.251	1.17E-02		**N:** 114<br>**UQ:** 3.142<br>**M:** 2.096<br>**LQ:** 1.160	**N:** 116<br>**UQ:** 0.463<br>**M:** 0.222<br>**LQ:** 0.065	2.2E-15	
	**RFS probability (10 years)**	**Upper quartile survival**				**Upper quartile survival**			
		**High expression (months)**	**Low Expression (months)**	**HR**	**P**	**High expression (months)**	**Low expression (months)**	**HR**	**P**
		53.56	42	0.8	9.7E-5	32	18.17	0.68	0.01
	**OS probability (10 years)**	**Upper quartile survival**				**Upper quartile survival**			
		**High expression (months)**	**Low Expression (months)**	**HR**	**P**	**High expression (months)**	**Low expression (months)**	**HR**	**P**
		NA	NA	0.68	0.001	NA	NA	0.46	0.01
**HRH4**	**Expression (TPM)**	**Normal tissue**	**Primary tumor**	**P**		**Normal tissue**	**TNBC**	**P**	
		**N:** 114<br>**UQ:** 0.093<br>**M:** 0.07<br>**LQ:** 0.035	**N:** 1097<br>**UQ:** 0.046<br>**M:** 0.025<br>**LQ:** 0.01	<1E-12		**N:** 114<br>**UQ:** 0.093<br>**M:** 0.07<br>**LQ:** 0.035	**N:** 116<br>**UQ:** 0.032<br>**M:** 0.021<br>**LQ:** 0.008	1.2E-13	
	**RFS probability (10 years)**	**Upper quartile survival**				**Upper quartile survival**			
		**High expression (months)**	**Low Expression (months)**	**HR**	**P**	**High expression (months)**	**Low Expression (months)**	**HR**	**P**
		66	38	0.67	2.1E-12	27	17.76	0.64	0.0006
	**OS probability (10 years)**	**Upper quartile survival**				**Upper quartile survival**			
		**High expression (months)**	**Low Expression (months)**	**HR**	**P**	**High expression (months)**	**Low Expression (months)**	**HR**	**P**
		NA	NA	0.86	0.21	80.64	56.4	0.7	0.16

Expression data were obtained from the UALCAN interactive web resource (http://ualcan.path.uab.edu/). The samples used for the analysis come from the genomic data of the project The Cancer Genome Atlas (TCGA). **N**: number of samples (patients); **UQ**: upper quartile; **M**: median; **LQ**: lower quartile. **NA**: Not available. Survival data were obtained from the portal-web Kaplan-Meier Plotter (http://kmplot.com/analysis/). The samples used for the analysis come from the Gene Expression Omnibus (GEO) and the "Cartes d'Identité des Tumeurs (CIT)" breast cancer (BRCA) datasets. Upper quartile survival to high and low expression of HDC or HRH4 in months, the hazard ratio (HR) and log-rank P value are shown.

Supplementary Figure 1

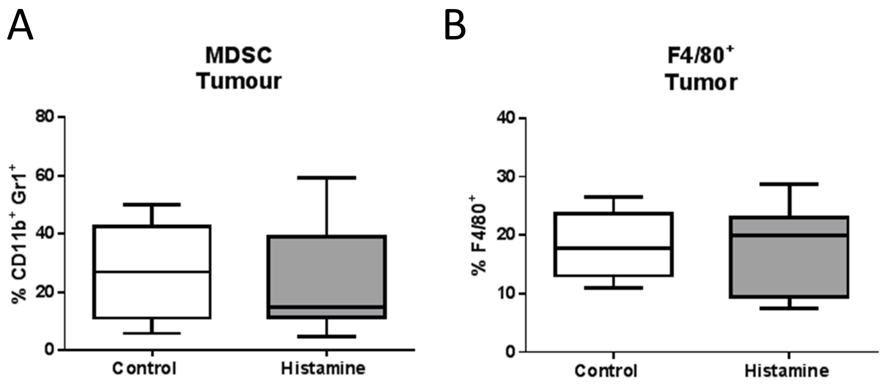

Supplementary Figure 1: Effect of histamine on the distribution of macrophages and myeloid-derived suppressor cells (MDSC) in 4T1 tumors. 4T1 tumour-bearing mice were left untreated (control) or were treated with histamine (5 mg kg^{-1}). Tumour cell suspensions were labelled with specific antibodies and determinations were performed by flow cytometry. **(A)** CD11b PE antibody and Ly-6G-Ly-6C FITC antibody: MDSC markers (n=16 mice) **(B)** F4/80 PE antibody: macrophage marker. To prevent non-specific binding of IgG to the FcγIII and FcγII receptors prior to staining with antigen specific primary antibodies, we preincubated cell suspensions with mouse BD Fc Block purified anti-mouse CD16/CD32 monoclonal antibody (BD Pharmingen) at 4°C for 5 minutes (n=10 mice). The box plots represent the median and interquartile range for each experimental group (Mann Whitney test, NS).

Supplementary Figure 2

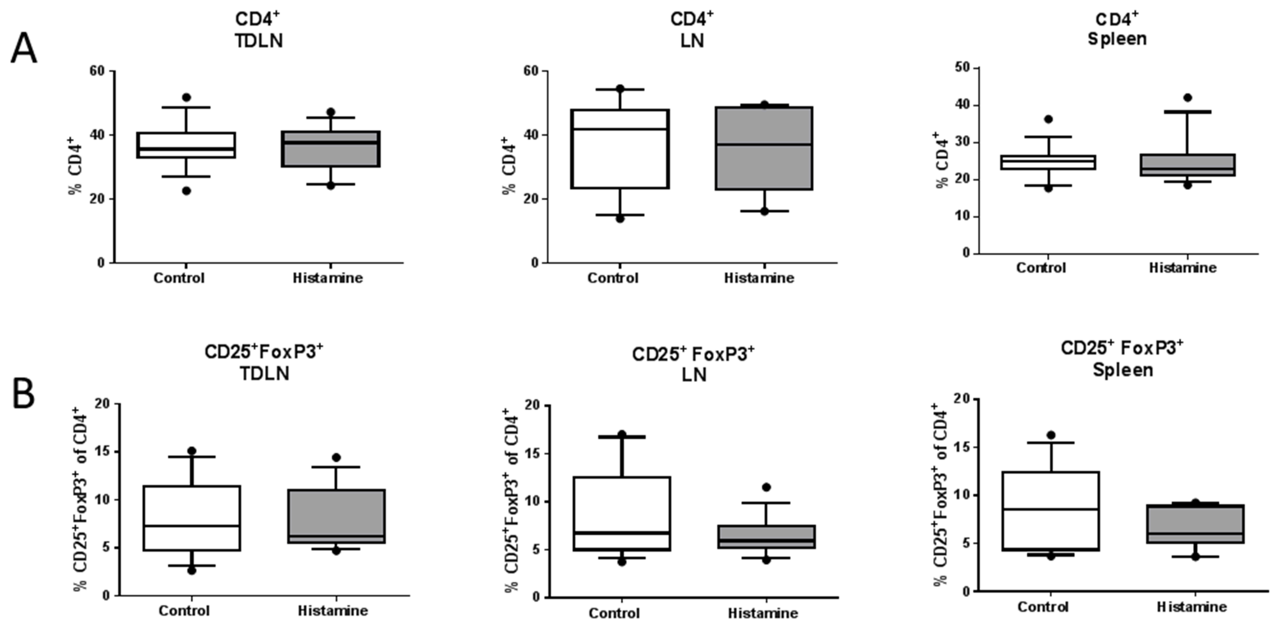

Supplementary Figure 2: Effect of histamine on immune cell subset distribution in spleens, tumour draining lymph nodes (TDLN) and non-draining lymph nodes (LN) of 4T1 tumour bearing mice. 4T1 tumour-bearing mice were left untreated (control) or were treated with histamine (5 mg kg^{-1}). **(A)** Spleen and lymph node cell suspensions were labelled with the CD4 FITC antibody (T helper lymphocytes marker) and the percentage of $CD4^{+}$ cells was determined by flow cytometry. **(B)** Within the gate of $CD4^{+}$ cells, the percentage of $CD25^{+}$ $FoxP3^{+}$ T regulatory cells was determined. Lines represent the median of n=10 mice. (Mann Whitney test, NS).

Supplementary Figure 3

A

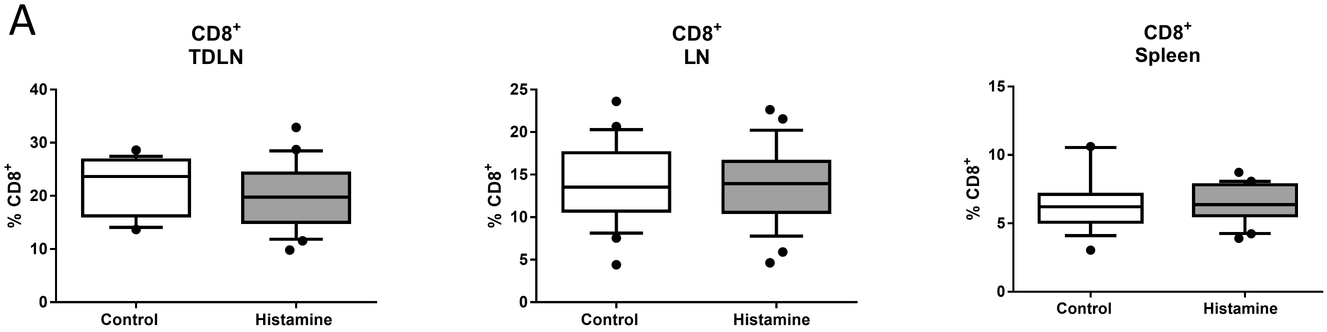

B

TDLN	Control	Histamine	P
% Lysis	21.4±1.0	19.2±2.2	NS
**LN**	Control	Histamine	P
% Lysis	25.2±3.2	20.7±2.8	NS
**Spleen**	Control	Histamine	P
% Lysis	20.5±1.0	23.9±3.3	NS

Supplementary Figure 3: Effect of histamine on CD8⁺ immune cell subset distribution and activity in spleens, tumour draining lymph nodes (TDLN) and non-draining lymph nodes (LN) of 4T1 tumour bearing mice. 4T1 tumour-bearing mice were left untreated (control) or were treated with histamine (5 mg kg^{-1}). **(A)** Spleen and lymph node cell suspensions were labelled with the CD8-PE antibody (T cytotoxic lymphocytes marker) and the percentage of $CD8^+$ cells was determined by flow cytometry. Lines represent the median of n=10 mice. **(B)** Splenocytes and lymph nodes cells from both treated and untreated mice were *ex vivo* stimulated with 4T1 irradiated cells for 5 days and then co-incubated for 4 h with CFSE-stained 4T1 cells and further staining with propidium iodide. The quantification of double positive cells was used as indicator of 4T1 cell lysis as a consequence of specific cytotoxic activity of T cells (n=4). (Mann-Whitney test, NS).

Supplementary Figure 4

A

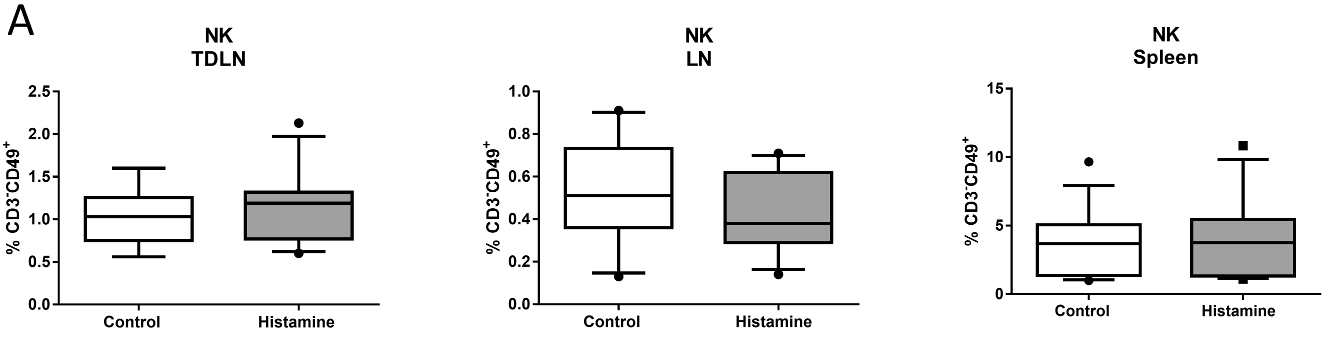

B

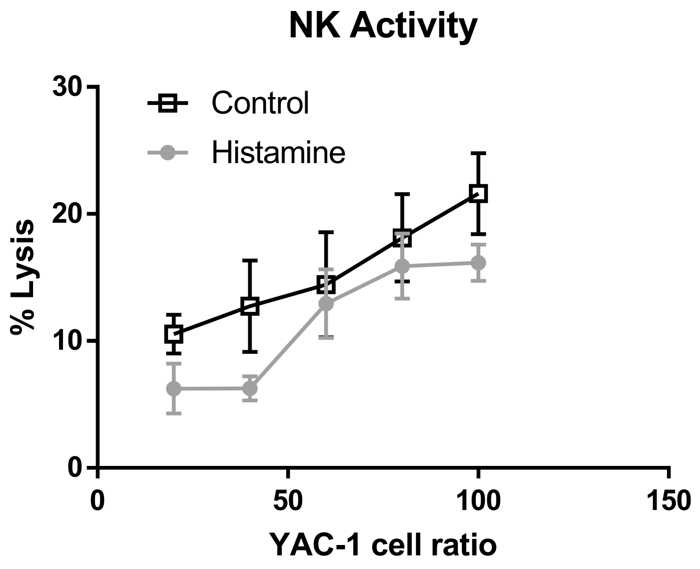

Supplementary Figure 4: Effect of histamine on NK immune cell subset distribution and activity in spleens, tumour draining lymph nodes (TDLN) and non-draining lymph nodes (LN) of 4T1 tumour bearing mice. 4T1 tumour-bearing mice were left untreated (control) or were treated with histamine (5 mg kg^{-1}). **(A)** Spleen and lymph node cell suspensions were labelled with the CD49b-PE antibody (NK cells marker) and the percentage of $CD49b^{+}$ cells was determined by flow cytometry. Lines represent the median of n=10 mice. **(B)** The NK cytotoxic activity of splenic cell suspensions was evaluated by co-incubating splenocytes with different ratios of CFSE-labelled YAC-1 cells for 4 h and further staining with propidium iodide. The percentage of double positive cells was used as an indicator of NK cytotoxic activity (n=3). (Mann-Whitney test, NS).
